# MutS*β* exceeds MutS*α* in dinucleotide loop repair

**DOI:** 10.1038/sj.bjc.6605531

**Published:** 2010-02-16

**Authors:** J Kantelinen, M Kansikas, M K Korhonen, S Ollila, K Heinimann, R Kariola, M Nyström

**Affiliations:** 1 Department of Biological and Environmental Sciences, University of Helsinki , P.O. Box 56 (Viikinkaari 5), Helsinki FI-00014, Finland; 2 Department of Genetics, University of Helsinki , P.O. Box 56 (Viikinkaari 5), Helsinki FI-00014, Finland; 3 Research Group Human Genetics, Department of Biomedicine, University of Basel, Basel CH-4005, Switzerland

**Keywords:** functional analysis, HNPCC, mismatch repair, MutS*α*, MutS*β*, MSH3

## Abstract

**Backround:**

The target substrates of DNA mismatch recognising factors MutS*α* (MSH2+MSH6) and MutS*β* (MSH2+MSH3) have already been widely researched. However, the extent of their functional redundancy and clinical substance remains unclear. Mismatch repair (MMR)-deficient tumours are strongly associated with microsatellite instability (MSI) and the degree and type of MSI seem to be dependent on the MMR gene affected, and is linked to its substrate specificities. Deficiency in *MSH2* and *MSH6* is associated with both mononucleotide and dinucleotide repeat instability. Although no pathogenic *MSH3* mutations have been reported, its deficiency is also suggested to cause low dinucleotide repeat instability.

**Methods:**

To assess the substrate specificities and functionality of MutS*α* and MutS*β* we performed an *in vitro* MMR assay using three substrate constructs, GT mismatch, 1 and 2 nucleotide insertion/deletion loops (IDLs) in three different cell lines.

**Results:**

Our results show that though MutS*α* alone seems to be responsible for GT and IDL1 repair, MutS*α* and MutS*β* indeed have functional redundancy in IDL2 repair and in contrast with earlier studies, MutS*β* seems to exceed MutS*α*.

**Conclusion:**

The finding is clinically relevant because the strong role of MutS*β* in IDL2 repair indicates MSH3 deficiency in tumours with low dinucleotide and no mononucleotide repeat instability.

The five proteins involved in the human mismatch repair (MMR) mechanism to maintain genomic integrity function as heterodimers are MutL*α* (MLH1+PMS2), MutS*α* (MSH2+MSH6) and MutS*β* (MSH2+MSH3). MMR proteins correct base/base mismatches and small insertion/deletion loops (IDLs) that arise on the newly synthesised strand during DNA replication and recombination. Larger loop structures (⩾5 nt) are believed to require a different combination of repair proteins and hence are not targets of the MMR mechanism (Umar *et al*, 1998). Approximately 25% of sporadic colon tumours, as well as a number of tumours of endometrium, ovary and some other organs and tissues, are deficient in MMR (Peltomäki, 2003). Moreover, germline mutations in MMR genes predispose to hereditary nonpolyposis colorectal cancer (HNPCC) syndrome/Lynch syndrome. To date, 659 *MLH1* (44% of all identified MMR gene variations), 595 *MSH2* (39%), 216 *MSH6* (14%) and 45 *PMS2* (3%) germline variations have been reported in the database (Woods *et al*, 2007; http://www.med.mun.ca/MMRvariants/). However, no HNPCC predisposing *MSH3* mutations have yet been identified.

MMR-deficient tumours are strongly associated with microsatellite instability (MSI) (Aaltonen *et al*, 1993). However, the degree and type of MSI differ from high to low and between mono-, di-, tri- and tetranucleotide instability or elevated microsatellite alterations at selected tetranucleotide repeats (EMAST) (Peltomäki and Vasen, 2004; Plaschke *et al*, 2004; Haugen *et al*, 2008) depending on the MMR gene affected. MLH1- and MSH2-deficient tumours are characterised by both mono- and dinucleotide repeat instability, whereas the level of MSI is lower in MSH6-deficient tumours (Bhattacharyya *et al*, 1995; Papadopoulos *et al*, 1995). MSH6-deficient cells are unable to repair single base mismatches, whereas they retain proficiency to repair two, three and four base loops (Drummond *et al*, 1995; Risinger *et al*, 1996; Umar *et al*, 1997), thus, causing only mononucleotide repeat instability in tumours (Wagner *et al*, 2001; Plaschke *et al*, 2004). Recently, EMAST and also low dinucleotide repeat instability have been associated with MSH3 deficiency both in tumour cell lines and in sporadic colorectal tumours (Haugen *et al*, 2008).

The type of MSI seems to be dependent on the substrate specificities of the MMR protein affected. In human cells, the MMR process is initiated by the binding of the mismatch recognition factor MutS*α* or MutS*β* to the mispair, followed by the initiation of the assembly of the repairosome by MutL*α* (Constantin *et al*, 2005; Zhang *et al*, 2005). MutS*β* has a high binding affinity to IDLs but, in contrast, a very low affinity to simple base/base mispairs (Acharya *et al*, 1996; Palombo *et al*, 1996), whereas MutS*α* has been shown to bind and repair both base/base mispairs and IDLs (Drummond *et al*, 1995; Palombo *et al*, 1996). Lesion specificity is believed to lie within the *MSH3/MSH6*-specific sequences, which differ notably (Owen *et al*, 2009). The process through which ADP–ATP exchange occurs on MSH2 seems to be dependent on the protein it forms a complex with; MSH6 requires ATP stabilisation, whereas MSH3 requires ATP hydrolysis, both of which are dependent on specific lesion binding (Owen *et al*, 2009). However, findings based on assays analysing the binding properties of these MMR proteins do not yet prove their functional ability to repair the bound mismatches (Ou *et al*, 2007).

In this study, we applied the *in vitro* MMR assay to analyse the substrate specificities and functionality of MutS*α* and MutS*β* using substrates, GT, IDL1 and IDL2 in three different cell lines. The *in vitro* MMR assay allows the functional analysis of all different MMR protein complexes and all kinds of missense variations in individual genes in a homologous human MMR system. In this study, the assay was for the first time applied to test the interference of an *MSH3* variation with repair efficiency.

## Materials and methods

### Cell lines and nuclear extracts

Cancer cell lines HeLa, LoVo, HCT116 (American Type Culture Collection, Manassas, VA, USA) and GP5d (European Collection of Cell Cultures, Salisbury, UK) were cultured according to instructions of manufacturers. HeLa cells are MMR proficient, whereas HCT116, LoVo and GP5d cells are MMR deficient. HCT116 cells lack MLH1 and MSH3 (*MSH3* is mutated as a consequence of the primary MMR defect) (Cannavo *et al*, 2005), whereas in LoVo cells, the *MSH2* gene is inactivated causing a deficiency of MSH2, MSH3 and MSH6 proteins (Drummond *et al*, 1997). The lack of MSH2 has been associated with the proteolytic degradation of its counterparts MSH3 and MSH6 (Cannavo *et al*, 2005). GP5d cells are MMR deficient because of primary alterations in *MSH2* and *MLH3*, resulting in lack of MSH6 and MSH3 proteins as well (Cannavo *et al*, 2005; di Pietro *et al*, 2005).

Nuclear proteins were extracted as described earlier (Lahue *et al*, 1989; Holmes *et al*, 1990). Approximately 2–10 × 10^8^ cells were collected and treated with 30–40 ml of cold isotonic buffer (20 mM Hepes pH 7.5, 5 mM KCl, 1.5 mM MgCl, 250 mM sucrose, 0.2 mM PMSF, 1 × complete EDTA-free protease inhibitor mixture (Roche Diagnostics GmbH, Mannheim, Germany), 0.25 *μ*g ml^−1^ aprotinin, 0.7 *μ*g ml^−1^ pepstatin, 0.5 *μ*g ml^−1^ leupeptin, 1 mM DTT). The cells were resuspended in cold hypotonic buffer (isotonic buffer without sucrose) followed by immediate pelleting. Approximately 1 ml per 1–2 × 10^8^ cells of hypotonic buffer was used to disrupt the cell membranes with a syringe and a narrow-gauge needle. Nuclei were collected by centrifugation and suspended in cold extraction buffer (25 mM Hepes pH 7.5, 10% sucrose, 1 mM PMSF, 0.5 mM DTT, 1 *μ*g ml^−1^ leupeptin) and NaCl up to 155 mM by rotation in +4°C for 1 h. The supernatant was dialysed for 2 h against cold dialysis buffer (25 mM Hepes pH 7.5, 50 mM KCl, 0.1 mM EDTA pH 8, 10% sucrose, 1 mM PMSF, 2 mM DTT, 1 *μ*g ml^−1^ leupeptine) and collected after further centrifugation.

### Preparation of heteroduplex molecules

The heteroduplex DNA molecule is a circular 3193 bp long molecule with a single-strand nick 445 bp upstream from the site of the mismatch. Three different heteroduplex constructs were prepared; a G–T mismatch (5′GT), and a single and two nucleotide IDLs (5′IDL1, 5′IDL2). Site-directed mutagenesis was carried out according to manufacturer's instructions (QuickChance Site-directed mutagenesis, Stratagene, La Jolla, CA, USA) to create the 1 nt (delA) and 2 nt (delAT) deletions to the positive pGEM IDL40 plasmid strand at the *Bgl*II restriction site. The GT mismatch was created by replacing adenine with guanine maintaining a thymine on the complementary strand. Single-stranded DNA was prepared by infecting pGEM IDL40 transformed XL1-blue bacteria cells with the M13K07 bacteriophage (Amersham Biosciences, Piscataway, NJ, USA), which replicates the antisense strand. Single-stranded DNA was extracted from the bacteriophages and used in excess to re-anneal with the linearised plasmid DNA creating heteroduplex molecules. Plasmid-safe DNAse and BND cellulose treatments were carried out to purify the final product.

### Production of wild-type heterodimer protein complexes

*Spodoptera frugiperda* (*Sf9*) (Invitrogen, Carlsbad, CA, USA) insect cells were transfected with bacmid DNA carrying wild-type (WT) *MSH2, MSH3, MSH6, PMS2* or *MLH1* cDNA fragments. The cells were then re-infected to obtain a higher yield of recombinant baculoviruses (Nyström-Lahti *et al*, 2002). WT-recombinant baculoviruses were used to co-infect *Sf9* cells for protein production forming the heterodimer complexes assayed: MutL*α* (MLH1+PMS2), MutS*α* (MSH2+MSH6) and MutS*β* (MSH2+MSH3). The heterodimeric complexes were extracted as total protein extracts (TE) at 50 h (MutL*α*) or 72 h (MutS*α* and MutS*β*) as described earlier (Kariola *et al*, 2002; Nyström-Lahti *et al*, 2002; Raevaara *et al*, 2004; Ollila *et al*, 2006).

### *MSH3* mutagenesis

The *MSH3* missense mutation (c.2386 C>T, RefSeq NM 002439.2) was constructed with a PCR-based site-directed mutagenesis kit according to manufacturer's instructions (QuickChance Site-directed mutagenesis, Stratagene) substituting arginine with tryptophan in codon 796 (p.R796W). The mutated *MSH3* cDNA was introduced into a pFastBac1 vector (Invitrogen) and sequenced (ABIPrism 3100 Genetic Analyzer; Applied Biosystems, Foster City, CA, USA). The primer sequences and PCR parameters are available on request. Proteins were produced and extracted from *Sf9* cells as described in the previous paragraph.

### Western blot analysis

Protein expression levels in the nuclear extracts (NEs) were studied by western blot analysis using 50 *μ*g of NE and 0.1–5 *μ*l of WT-TE by means of sodium dodecyl sulphate polyacrylamide gel electrophoresis. The proteins were blotted into nitrocellulose membranes (Hypond, PVDF, Amersham Pharmacia Biotech, Uppsala, Sweden), which were subsequently incubated with monoclonal antibodies anti-MSH2 (Calbiochem, San Diego, CA, USA, MSH2-Ab1, NA-26, 0.2 *μ*g ml^−1^), anti-MSH3 (BD Transduction Laboratories, Lexington, KY, USA, M94120, 250 *μ*g ml^−1^), anti-MSH6 (BD Transduction Laboratories, clone 44, 0.02 *μ*g ml^−1^), anti-PMS2 (Calbiochem/Oncogene Research, San Diego, CA, USA, Ab-1, 0.2 *μ*g ml^−1^) and anti-MLH1 (BD Biosciences/Pharmingen, San Diego, CA, USA, clone 168-15, 0.5 *μ*g ml^−1^). Ubiquitously expressed *α*-tubulin was used as a loading control to estimate the MMR protein levels in the extracts (anti-*α*-tubulin; Sigma, Louis, MO, USA, DM1A, 0.2 *μ*g ml^−1^).

### The *in vitro* MMR assay

**Figure 1:**
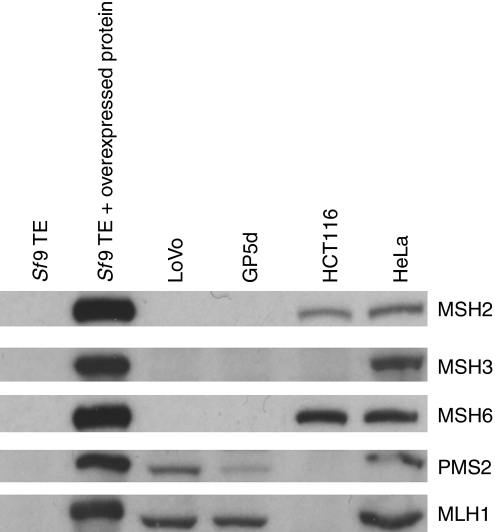
Western blot analysis of the MMR protein contents in the NEs used in the functional assay. HeLa, a positive control, includes all five MMR proteins, MLH1, PMS2, MSH2, MSH3 and MSH6. HCT116 lacks MLH1, PMS2 and MSH3. Both GP5d and LoVo lack MSH2, MSH3 and MSH6. As an assay control, *Sf9* TE are included with and without the overexpressed WT MMR proteins. The loading control, *α*-tubulin is not shown.

The roles of MutS*α*-WT and MutS*β*-WT in 5′GT/5′IDL1/5′IDL2 repair were analysed by an *in vitro* MMR assay as described earlier (Nyström-Lahti *et al*, 2002). Repair reactions were standardised to include 75–100 *μ*g of MMR-deficient NE (HCT116, LoVo or GP5d), or an equal amount of MMR-proficient HeLa extract. The excess amount of the heteroduplex DNA substrate (5′GT, 5′IDL1 or 5′IDL2) was set to 100 ng. The functionality of WT-proteins was assayed by complementing HCT116, LoVo and GP5d NEs with an appropriate amount of *Sf9* total extract including the overexpressed MutL*α*-WT (50 ng), MutS*α*-WT (200 ng) or MutS*β*-WT (600 ng). The amount of WT-protein in the reaction was titrated to obtain maximum repair efficiency in each cell line. Owing to the low PMS2 expression, GP5d NE was complemented with MutL*α*-WT in each reaction (Figure 1). MMR-proficient HeLa NE was used as a positive control, whereas uncomplemented NEs as well as extracts complemented with untransfected *Sf9* proteins were used as negative controls. The substrates were linearised with *Eco31*I restriction enzyme. As the repair reaction converts a GT heteroduplex to an AT homoduplex or fills the 1 or 2 nt loop structures recreating the *Bgl*II restriction site, the repair efficiency can be measured by the efficiency of the double restriction. The functionality of mutated MSH3 TE was studied using 5′IDL2 substrate and GP5d NE. Repair percentages were analysed using Image-Pro 4.0 (Media Cybernetics, Silver Spring, MD, USA) and calculated as an average of three independent experiments. Statistical *t*-test analysis was carried out to evaluate the significance of the percentage differences observed between MutS*α* and MutS*β* in IDL2 repair.

## Results

### MMR protein contents of the cell lines used in the *in vitro* MMR assay

Western blot analysis was used to characterise the MMR protein contents in the cell lines used in the functional assay. The analysis confirmed the absence of MSH2, MSH6 and MSH3 in both LoVo and GP5d NEs, thus making them suitable for substrate specificity and functionality studies of MutS*α* and MutS*β* (Figure 1). Owing to the significantly reduced level of PMS2 in GP5d NE, together with the MutS complex, it was complemented with MutL*α*-WT. HCT116 NE was shown to express only MSH6 and MSH2 presenting an opportunity to study the substrate specificity and repair efficiency of MutS*β* (MSH3) and MutL*α*. The presence of all five MMR proteins in HeLa NE establishes its aptitude for functioning as a positive control.

### The *in vitro* MMR assay elucidates the substrate specificities and repair efficiencies of MutS*α* and MutS*β*

**Figure 2:**
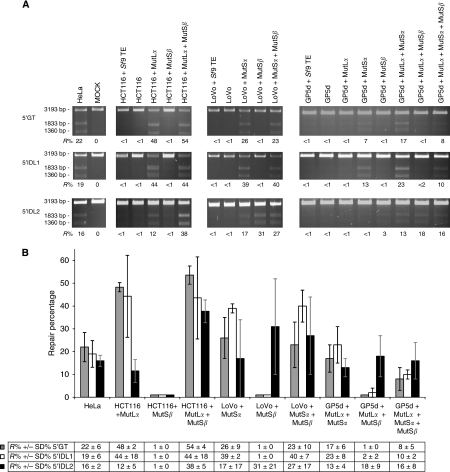
MMR efficiency of HCT116, LoVo and GP5d NEs complemented with MutS*α*, MutS*β* and MutL*α* complexes for 5′GT, 5′IDL1 and 5′IDL2 substrates. (**A**) Mock represents heteroduplex only, with no added NE or recombinant protein. MMR-proficient HeLa NE including all five MMR proteins is used as a positive control. MMR-deficient HCT116, LoVo and GP5d NEs and NEs complemented with *Sf9* insect cell TE are used as negative controls. The top fragment (3193 bp) represents the unrepaired linearised heteroduplexes and the two lower fragments (1833 and 1360 bp) show the migration of the repaired and double-digested DNA molecules. The repair percentages (*R*%) represent fractions of repaired DNA calculated as a ratio of double-digested DNA relative to total DNA added to the reaction. Values are a mean of three independent experiments. (**B**) The comparison of substrate-specific repair efficiencies of the MMR protein complexes (repair efficiency *R*% and s.d.±%). MutS*α* is able to repair all three substrates (5′GT/5′IDL1/5′IDL2), whereas MutS*β* does not repair 5′GT or 5′IDL1 in any extracts. However, complementation of HCT116 NE (lacking MLH1, PMS2 and MSH3) with MutL*α* alone yields a considerably lower IDL2 repair percentage (12%, s.d.±5%) than after co-complementation with MutL*α* and MutS*β* (38%, s.d.±5%). Moreover, complementation of LoVo and GP5d NEs (lacking MSH2, MSH3 and MSH6) with MutS*α* yields lower IDL2 repair percentages, 17% (s.d.±17%) and 13% (s.d.±4%), than when complemented with MutS*β*, 31% (s.d.±21%) and 18% (s.d.±9%), respectively.

With the right selection of cell lines, the *in vitro* MMR assay allows the functional analysis of all different MMR protein complexes in a homologous human MMR system. Here, three different substrates, 5′GT, 5′IDL1 and 5′IDL2 were used to study the substrate specificities and repair efficiencies of MutS*α* and MutS*β*. In contrast with MutL*α*, the presence of which is known to be vital for all these substrates, the type of MutS complex required for optimal repair efficiency is determined by the substrate construct. The MMR assay with LoVo, GP5d and HCT116, with various combinations of natural or complemented MutL*α* demonstrated that the role of MutS*α* is evident in the repair of 5′GT and 5′IDL1, whereas the repair of dinucleotide loops requires MutS*β* for efficient repair (Figure 2). Remarkably, all three cell lines demonstrate more efficient dinucleotide repair with MutS*β* than with MutS*α*. In HCT116, the mean repair efficiency was 26% higher with MutS*β* than with MutS*α* (*P*=0.0014), in LoVo it was 14% higher (*P*=0.284) and in GP5d 5% higher (*P*=0.230).

### The functional analysis of an *MSH3* variation

**Figure 3:**
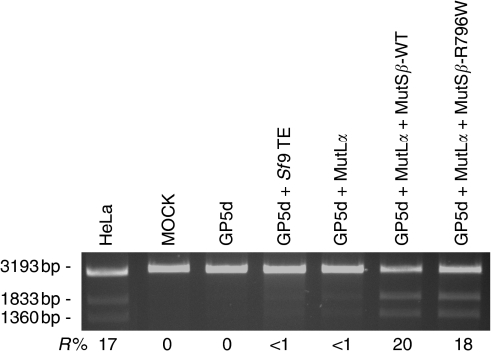
The functional analysis of MSH3-R796W. Both MutS*β*-WT (20%, s.d.±5%) and MutS*β*-R796W (18%, s.d.±8%) restore the repair capability of GP5d+MutL*α*. The values indicated are averages obtained from three separate experiments.

The *in vitro* MMR assay allows the functional analysis of all kinds of missense variations of the five different MMR genes. The strong role of MutS*β* (MSH2+MSH3) in dinucleotide loop repair and the use of LoVo and GP5d NEs, which lack both MutS*α* and MutS*β*, allow the functional analysis of MSH3 variations in a manner excluding false positives resulting from the presence of MutS*α*. In this study, for the first time, the assay was applied to test the effect of an *MSH3* variation (MSH3-R796W) on its repair efficiency. The variation was found in a putative HNPCC family (unpublished). Our result suggests that the mutated MSH3-R796W protein is proficient with repair percentages of MSH3-WT 20% (s.d.±5%) and MSH3-R796W 18% (s.d.±8%) (*P*=0.358) (Figure 3).

## Discussion

Using different substrate structures, the *in vitro* MMR assay is able to demonstrate differences in substrate specificities as well as in repair efficiencies of MutS*α* and MutS*β*. The overlapping roles of these heterodimeric complexes have been reported earlier (Acharya *et al*, 1996; Genschel *et al*, 1998) generally emphasising the role of MutS*α* predominantly for the recognition of base/base mispairs and small IDLs and MutS*β* for the recognition of larger (>2 bp) IDLs (Acharya *et al*, 1996; Palombo *et al*, 1996). Our experiments support the functional redundancy but contradictory to the previous impression, in this study, the repair efficiency of MutS*β* was shown to exceed that of MutS*α* in the repair of dinucleotide loop structures.

The lack of a functional MMR mechanism causes MSI. HCT116 cells, which are deficient in MLH1 and MSH3, complemented with *MLH1* through the addition of chromosome 3 have been shown to demonstrate mononucleotide repeat stability but still a low level of dinucleotide and a high level of tetranucleotide repeat instability suggesting a problem in *MSH3*. Although, the tetranucleotide repeat markers represented a level of instability five times higher than dinucleotide repeats supporting the functional overlap of MutS*β* and MutS*α* in IDL2 repair, low dinucleotide repeat instability was caused by defected MutS*β* (MSH3) (Haugen *et al*, 2008). The microsatellite stability was indeed reversible by complementing HCT116 cells with both chromosomes 3 and 5, hence expressing both lost proteins MLH1 and MSH3 (Haugen *et al*, 2008). In this study, MutS*β* not only participated in IDL2 repair but exceeded the repair efficiency of MutS*α* proven in three different cell lines, HCT116, LoVo and GP5d. Although, HCT116 expressed a sufficient amount of MutS*α* to repair GT and IDL1 mismatches, the repair efficiency of IDL2 increased three-fold when the cells were complemented with MutS*β*. An increase in repair efficiencies was also seen in LoVo and GP5d cells when complemented with MutS*β* but the differences of repair efficiencies between MutS*α* and MutS*β* were not statistically significant as in HCT116.

By selecting a cell line that lacks the analysed MMR protein, the *in vitro* MMR assay allows the functional analysis of all different MMR proteins and any missense mutation in an individual MMR gene. The assay has been applied earlier to a large number of *MSH2/6* and *MLH1* mutations using GT and IDL1 as target substrates (Nyström-Lahti *et al*, 2002; Kariola *et al*, 2004; Raevaara *et al*, 2005; Ollila *et al*, 2006). In this study, the finding of the strong role of MutS*β* (MSH2+MSH3) in IDL2 repair enables efficient testing of *MSH3* variations. By selecting LoVo and GP5d NEs, which lack both MutS*α* and MutS*β*, the assay was adapted to study the functional significance of an *MSH3* variation for the first time. Although the analysed variant was shown to be proficient in IDL2 repair, the assay itself functioned well suggesting its utility for further *MSH3* testing.

In cancer diagnostics, the MSI phenotype has been a hallmark of HNPCC tumours. However, the levels have varied from high to low or to no MSI and between mono-, di-, tri- and tetranucleotide repeat instability dependent on the MMR gene affected and its substrate specificities. Generally, the MSI marker panel used in the diagnostics includes mono- and dinucleotide markers, and in MSI-positive cases, *MLH1, MSH2* and *MSH6* genes associated with mono- and dinucleotide repeat instability are analysed for mutations. Our results are clinically relevant emphasising the importance of MSH3 in dinucleotide loop repair and we encourage performing *MSH3* mutation analysis when a tumour shows dinucleotide but no mononucleotide repeat instability.
